# Profiling DNA methylation patterns of zebrafish liver associated with parental high dietary arachidonic acid

**DOI:** 10.1371/journal.pone.0220934

**Published:** 2019-08-09

**Authors:** Anne-Catrin Adam, Kai Kristoffer Lie, Paul Whatmore, Lars Martin Jakt, Mari Moren, Kaja Helvik Skjærven

**Affiliations:** 1 Institute of Marine Research, Bergen, Norway; 2 Faculty of Biosciences and Aquaculture, Nord University, Bodø, Norway; New England Biolabs Inc., UNITED STATES

## Abstract

Diet has been shown to influence epigenetic key players, such as DNA methylation, which can regulate the gene expression potential in both parents and offspring. Diets enriched in omega-6 and deficient in omega-3 PUFAs (low dietary omega-3/omega-6 PUFA ratio), have been associated with the promotion of pathogenesis of diseases in humans and other mammals. In this study, we investigated the impact of increased dietary intake of arachidonic acid (ARA), a physiologically important omega-6 PUFA, on 2 generations of zebrafish. Parental fish were fed either a low or a high ARA diet, while the progeny of both groups were fed the low ARA diet. We screened for DNA methylation on single base-pair resolution using reduced representation bisulfite sequencing (RRBS). The DNA methylation profiling revealed significant differences between the dietary groups in both parents and offspring. The majority of differentially methylated loci associated with high dietary ARA were found in introns and intergenic regions for both generations. Common loci between the identified differentially methylated loci in F_0_ and F_1_ livers were reported. We described overlapping gene annotations of identified methylation changes with differential expression, but based on a small number of overlaps. The present study describes the diet-associated methylation profiles across genomic regions, and it demonstrates that parental high dietary ARA modulates DNA methylation patterns in zebrafish liver.

## Introduction

Methylation of the cytosine nucleotide, generally referred to as DNA methylation, is the most widely studied epigenetic mechanism. DNA methylation is crucial for regulating cell differentiation and development [1–3], and can be affected in response to environmental stimuli, such as nutrition [4–7]. Cytosine methylation plays a key role in transcriptional regulation, and can thereby influence physiological processes [8, 9]. Methylation primarily occurs in CpG sites where a cytosine is followed by a guanine. CpG islands are genomic regions highly enriched in CpG sequences, and often associated with promoter regions of a gene [10]. CpG methylation located in promoters [11, 12], first exons [13], gene bodies [14–16], or enhancer elements [17] of the genome can influence the transcriptional activity of neighbouring genes, though the mechanisms by which methylation affects transcription in other genomic regions is still largely unknown. Additionally, epigenetic patterns can be transmitted to the next generation through the germline or the developing embryonic environment [18], but to what degree nutrients in the parental diet influence gene regulation in the next generation is not fully understood.

Nutrition during early life stages can have an impact on health and disease disposition later in life [19–22]. Both maternal and paternal diets have been shown to influence egg production, viability and gene expression of the embryos in different model species [23–25]. As DNA methylation may be mitotically stable and heritable through the germline [26–29], dietary influence or other environmental exposures can potentially be ‘remembered’ in the epigenome and transmitted to the next generation [30]. It is conceivable that parental diet can influence gene expression potential in the progeny, especially under different environmental conditions, such as lifestyle and diet [31–33]. It has been shown in teleost species that dietary micronutrient levels given to the parents affected gene expression in embryo and fry [23, 33] as well as DNA methylation in livers of adult offspring [5].

High dietary intake of omega-6 (n-6) PUFAs, especially arachidonic acid (ARA, 20:4n-6) has evoked some concern due to their role in inflammatory processes and in relation to prevalence of certain diseases [34–37]. In fish, increased ARA in the diet has an impact on bone development [38, 39], growth and reproduction through improving hatching rate and accumulating ARA in ovary and eggs [40, 41]. PUFAs are capable of modulating DNA methylation patterns as shown in studies in mammals [42, 43], though studies in mice have observed no link between DNA methylation differences in adult progeny livers and the maternal high fat diet [44]. However, the latter study did show a strong effect of the maternal diet on the hepatic expression of genes directing to inflammation, cholesterol synthesis and RXR activation [44]. A study using human THP-1 monocytes revealed general DNA hypermethylation in response to ARA, and it was suggested that the changes in *β*-oxidation and PPAR-α were important mediators of ARA-induced DNA methylation changes [45]. Another study on human vascular endothelial cells suggested that ARA metabolism was sensitive to changes in DNA methylation, but whether these methylation changes affected other enzymes regulating the ARA metabolism was not known [46]. Although there are findings showing an effect of dietary fatty acids on methylation profiles, more studies are needed to investigate both mechanisms and how changes in DNA methylation can influence gene regulation in adult progeny.

Recently, we showed that parental high dietary ARA levels affected hepatic gene expression in the offspring, and levels of immune-related eicosanoids, lipids and oxidised metabolites in the first generation of zebrafish [47, 48]. In the present study, we used zebrafish as a nutritional model to investigate the effect of a parental high ARA diet on hepatic DNA methylation patterns in both parents and progeny using reduced representation bisulfite sequencing (RRBS). We compared our DNA methylation results with previously described transcriptomic profiles in the livers [48].

## Materials and methods

### Ethical considerations

Animal care and performance of the experimental trial conform to the principles of the Norwegian Animal Research Authority and the study was approved by the Norwegian Food Safety Authority (division no. 54, reference 2012/145126).

### Zebrafish feeding trial

**Fig 1:**
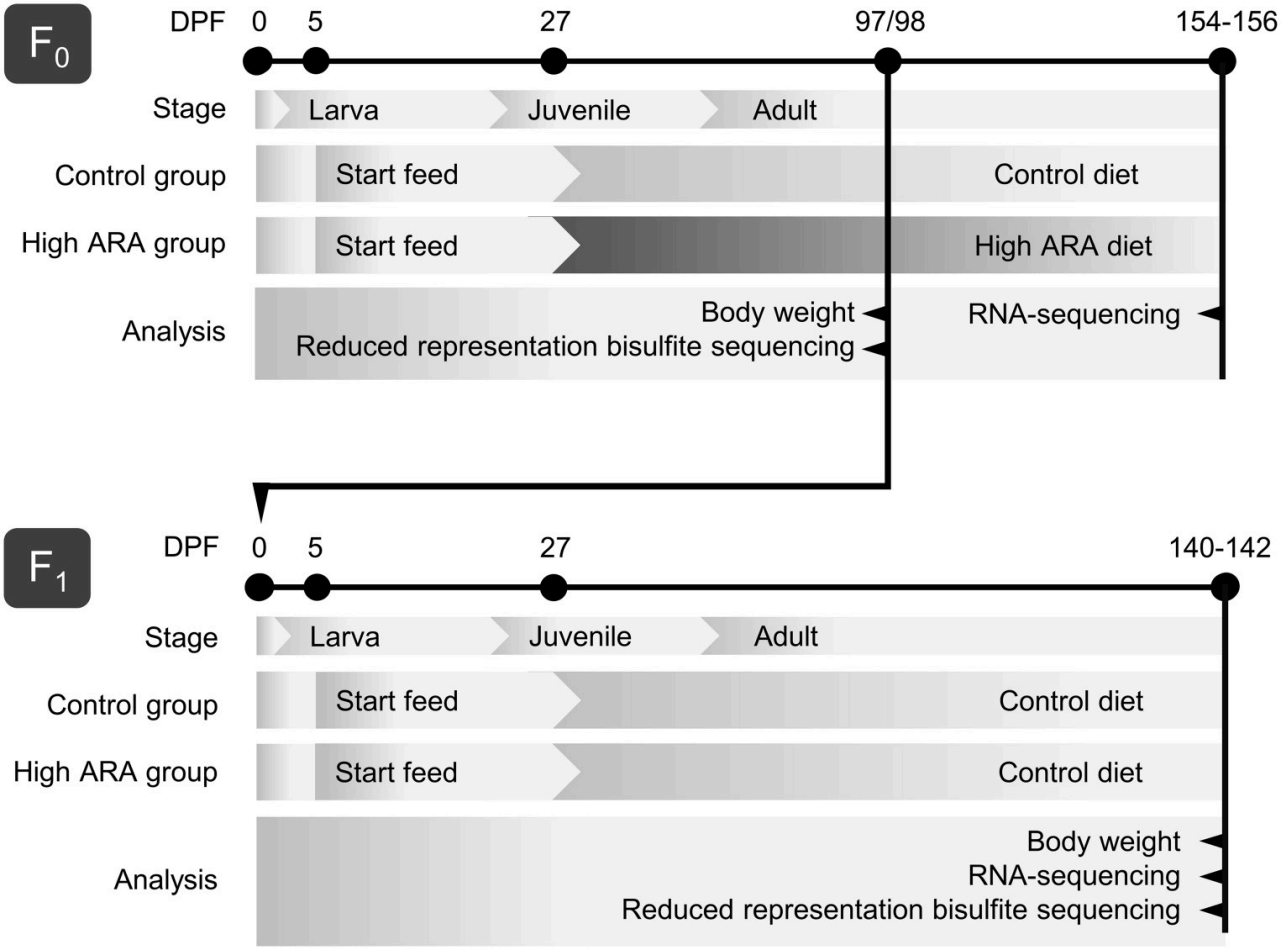
Setup of the intergenerational zebrafish feeding trial. Both generations of zebrafish were fed Gemma micro and *Artemia nauplii* as start feed from 5 and 7 days post fertilization (DPF) until 26 DPF, respectively. The experimental diet (control and high ARA) was given from 27 DPF until sampling for F_0_ only. Adult F_0_ were mated at 97 DPF to generate F_1_. Both F_1_ groups were fed the control diet from 27 DPF. Body weight and liver sampling for reduced representation bisulfite sequencing were performed at day 98 for F_0_ while at 140–142 DPF for F_1_.

Study design, zebrafish husbandry and standardized operating procedures for mating, handling and feeding for both F_0_ and F_1_ generation of wildtype AB zebrafish (*Danio rerio*) has been described previously [23]. In short, fish were kept in 10 gender mixed tanks (containing 60 fish each) per dietary group. F_0_ and F_1_ larvae were fed with Gemma micro (Skretting, Norway) and *Artemia nauplii* (Silver Star *Artemia*, USA) as start feed from 5 days post fertilization (DPF) and from 7 DPF until 26 DPF, respectively (Fig 1). The experimental diets were given twice a day from 27 DPF onwards. The F_0_ generation got either the control diet that was lower in ARA (1.87 mg ARA/g diet), or the high ARA diet (20.66 mg ARA/g diet). Diets were based on the requirement levels for carp [49]. Ingredients and nutritional composition of the diets are given in S1 File. F_0_ fish were mated at 97 DPF to receive F_1_ progeny. The F_1_ generation from both parental dietary groups (Control and high ARA group) were fed the control diet from 27 DPF onwards until liver dissection for DNA and RNA extraction.

### Liver tissue sampling

Mature zebrafish were deprived for food 18 h prior to sampling. They were anesthetized with 0.05% Tricaine Methane Sulphonate (PHARMAQ AS, Norway), blotted dry on tissue paper, weighed and euthanized by cutting the cardinal vein prior to liver dissection. Six single male livers (98 DPF (F_0_) and 140–142 DPF (F_1_)) from six tank populations (6n) of each dietary group were dissected, rinsed in 1x PBS, snap frozen with liquid nitrogen and stored at -80°C until DNA extraction. For RNA extraction, six pooled male livers from each of the six separate tanks from both groups were randomly sampled over two days between 154–156 DPF (F_0_) and 140–142 DPF (F_1_) due to simultaneous sampling for other analyses connected to this feeding trial.

### DNA and RNA extraction

Liver tissue was treated with RNase A (50ng/μL, 10 min at room temperature, Wizard SV Genomic DNA Purification System, Promega, USA) and proteinase K (20μg/μL, 1.5h at 55°C, NEB #P8102S, New England Biolabs (NEB), USA) prior to DNA extraction following the manufacturer’s instructions (Wizard SV Genomic DNA Purification System). DNA was eluted in nuclease-free water, quantity was measured using Qubit fluorometric quantitation (Life Technologies, USA) and extracted DNA was stored at -20°C.

Total RNA was extracted using QIAzol Lysis Reagent (Qiagen, Germany) and DNase treated with the Ambion^™^ DNA-free^™^ DNA Removal Kit (Invitrogen, USA). RNA quantity was measured using NanoDrop ND-1000 Spectrophotometer (NanoDrop Technologies, USA). RNA integrity (RIN), which was 9.06 ± 0.39 on average, was determined using an Agilent 2100 Bioanalyzer (RNA 6000 Nano LabChip kit, Agilent Technologies, USA).

### RRBS and DNA methylation calling

Reduced representation bisulfite sequencing (RRBS) was used to measure DNA methylation with single-base resolution, and to enrich amplified genomic regions [50]. RRBS and initial processing of the RRBS data was completed by the Biomedical Sequencing Facility (BSF, Vienna, Austria). RRBS library preparation was based on previous RRBS studies [51, 52]. Genomic DNA was digested by MspI (NEB #R0106L, 20 U/μL), followed by single-end preparation of the DNA fragments consisting of adapter ligation and A-tailing. Fragments were size-selected by performing a 0.75× cleanup with AMPure XP beads (A63881, Beckman Coulter, Inc, USA) for bisulfite conversion, enriched by PCR amplification and subsequent sequencing on an Illumina HiSeq 2000 platform in a 50/51bp single read mode [51]. FastQC software [53] was used for quality control of the sequences. Bisulfite reads were trimmed for low-quality and adapter sequences using a custom pipeline [52]. Bisulfite conversion metrics are given in S2 File. Reads were aligned to the Zebrafish Genome Assembly GRCz10 (danRer10) using BSMAP [54]. DNA methylation calls were performed using BiSeq [50]. Due to low DNA quality for sequencing of two samples and divergent sequencing results of one sample (all three samples were control F_0_), they were excluded from further downstream analysis. The data discussed has been stored in SRA (https://www.ncbi.nlm.nih.gov/bioproject/PRJNA418670) and is accessible through accession number PRJNA418670.

### Differential methylation and functional enrichment analysis

Differential methylation and downstream analysis was completed in R version 3.3.2 [55]. Methylation calls were filtered after a minimum read depth per locus of 10, and replicates were combined into a single table based on genomic loci that were present in all replicates and control samples to compare the same loci for differential methylation in all samples. One outlier replicate was removed after principal component analysis and pairwise sample distance plot inspection (dendrogram, heatmap). Differentially methylated loci (DML, differentially methylated cytosine in CpG context) were detected using the methylKit package, based on logistic regression analysis and Benjamini-Hochberg false discovery correction of p-values (q-values) [56]. Methylation events with a methylation difference +/- ≥25% and q-value ≤0.01 were considered for assigning hypermethylation (positive percentage) or hypomethylation (negative percentage) in the high ARA group compared to control. The ‘genomation’ package [57] was used to annotate DML by genomic regions such as promoters (±1000bp from the transcription start site), introns, exons, intergenic regions (gene nearest to the DML), CpG islands and CpG shores (±2000bp flanking regions around a CpG island). Definitions for genomic regions were obtained from the UCSC Genome Browser [58], which were coordinate mapped to GRCz10 Ensembl transcripts. Using a hypergeometric test in R, we also examined if genomic regions (CpG islands, CpG island shores, exons, introns and promoters) were significantly enriched or depleted (p<0.05) for DML. In addition, we calculated enrichment scores based on the ratio of DML to methylated loci within the genomic region (i.e. detected number of DML) divided by the ratio of total DML to total methylated loci within the entire genome (expected number of DML). Positive scores indicate the genomic region is enriched for DML (i.e. more DML detected than expected) and a negative score indicates depletion for DML (S3 File). Annotation of DML to gene identifiers such as Ensembl and Entrez gene identifier, gene symbol and gene description was completed using the genomicRanges [59] and biomaRt packages [60]. The annotated DML (S4 File) were used for functional annotation for KEGG pathways and GO terms by over-representation testing using the R package ‘clusterProfiler’ [61] (S5 File).

### Gene expression data

RNA-sequencing (RNA-seq) was performed by the Norwegian Sequencing Centre (NSC) doing the library preparation using TruSeq^™^ Stranded mRNA Library Prep Kit (Illumina, Inc, USA), and sequencing on the NextSeq500 platform (Illumina, Inc, USA) to generate single-end 75bp reads as previously reported [48]. Briefly, reads were mapped to the GRCz10 (Genome Reference Consortium Zebrafish Build 10) assembly based on Ensembl annotation data, [62] using the default parameters of HISAT2 [63]. Read counts per gene were quantified using featureCounts [64] and pre-filtered to exclude combined mean read counts smaller than 10. Differential gene expression was estimated using DESeq2 [65] and the complete list of expressed genes from F_0_ and F_1_ livers is reported in the S6 File. We identified significant differential expressed genes (DEG) using an adjusted p-value cut-off <0.05 for F_1_ DEG and <0.1 for F_0_ DEG due to fewer genes in the latter [48]. The gene expression data was obtained from a different cohort of zebrafish than the DNA methylation data, though the experimental treatment was the same as for the zebrafish in this present study. Raw data is accessible at the NCBI’s Gene Expression Omnibus [50] through GEO Series accession number GSE104692 (https://www.ncbi.nlm.nih.gov/geo/query/acc.cgi?acc=GSE104692).

DEG were converted to human orthologues to identify upstream regulators that can explain the observed gene expression changes using Ingenuity Pathway Analysis software suite (IPA, Ingenuity Systems, USA). Human orthologues were predicted using the OrthoRetriever v1.2 software. An overlap p-value using Fisher’s Exact test and an activation z-score (positive or negative) for upstream regulators is calculated based on prior knowledge stored in the Ingenuity Knowledge Base. Upstream regulators with missing z-scores and p >0.05 were filtered out from our analysis. Upstream regulators, which were only differentially methylated in F_0_, were also excluded from the final list. Positive and negative z-scores predict an activation and inhibition of an upstream regulator, respectively. Higher positive z-scores or lower negative z-scores indicate stronger directional relationship.

### Statistical analysis

Body weight of 98 DPF (F_0_, control: 36 fish, high ARA: 36 fish) and 140–142 DPF (F_1_, control: 48 fish, high ARA: 47 fish) zebrafish was initially tested for tank variances (10 tanks in F_0_ and 6 tanks in F_1_) using one-way ANOVA (GraphPad Prism 8, GraphPad Software, Inc, USA). An unpaired, two-tailed *t*-test (GraphPad Prism 8, GraphPad Software, Inc, USA) was used for significance testing (p-value <0.05) of body weight from all tank populations within a dietary groups given as mean ± standard deviation. For RRBS and RNA-seq, statistical treatment such as filtering, multiple comparison correction and hypergeometric test are described in previous respective sections. Downstream statistical analysis of RRBS and RNA-Seq data was completed using R v3.3.2 (http://cran.rproject.org/).

## Results

### Body weight

No significant differences in body weight were observed between the dietary groups of either F_0_ or F_1_. Mean body weight of F_0_ zebrafish was 0.31 ± 0.12 g in the control and 0.35 ± 0.13 g in the high ARA group. Mature F_1_ progeny body weight was on average 0.35 ± 0.04 g in the control and 0.34 ± 0.05 g in the high ARA group.

### Differential DNA methylation in F_0_ and F_1_ livers

#### General DNA methylation patterns

**Fig 2:**
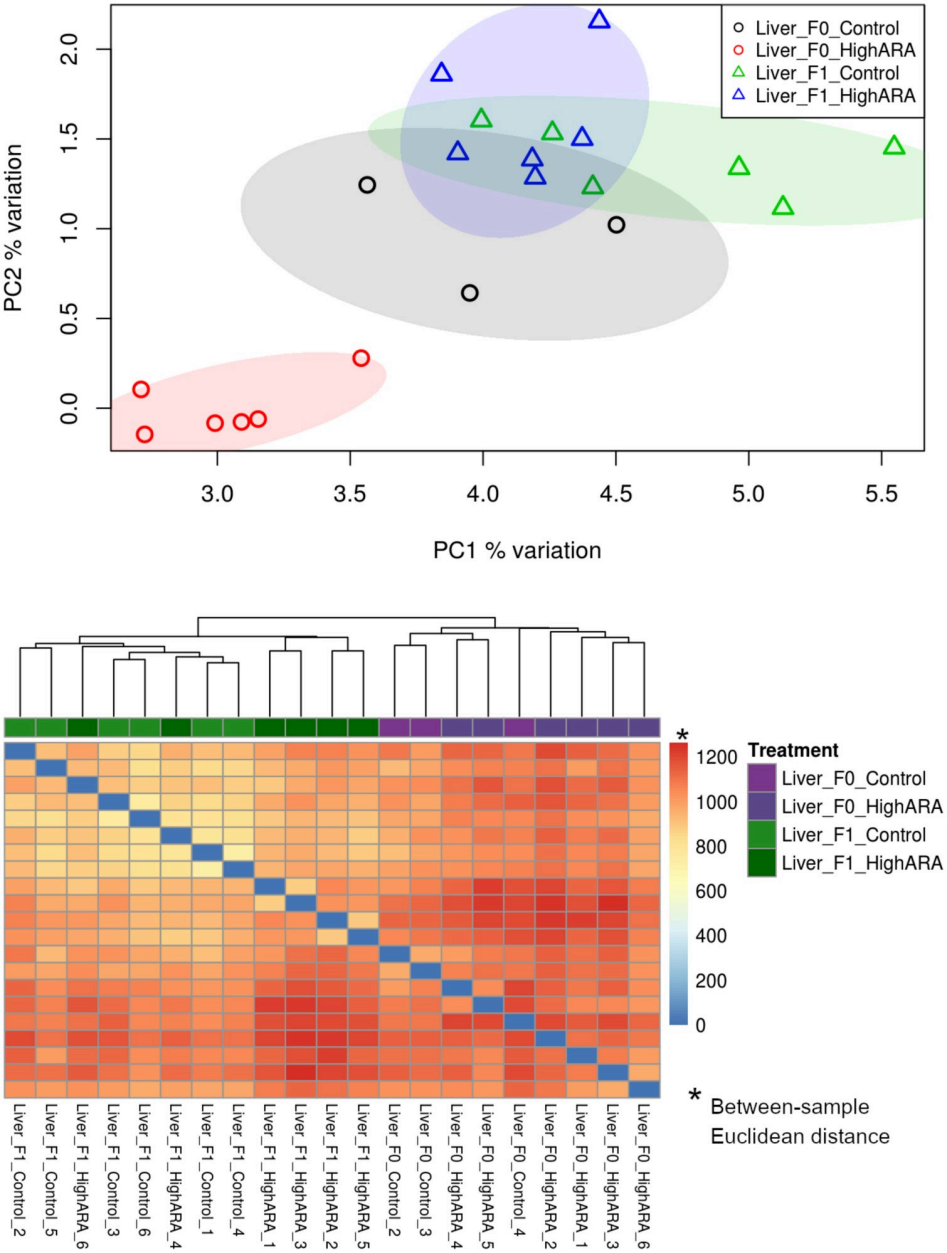
Clustering of livers from parents (F_0_) and progeny (F_1_) with regard to DNA methylation. Principal component analysis (A), and pairwise distance clustering of the samples (dendrogram and heatmap) (B) show the grouping of dietary groups based on percent methylation per locus in F_0_ livers from fish fed either the control or high ARA diet, and F_1_ fed the control diet. The scale for Fig 2B (0:1200) indicates Euclidean distance between samples as calculated by the base R package ‘dist’ (http://cran.rproject.org/).

An average of 84% of reads per sample were mapped to the zebrafish genome using BSMAP (S2 File). Bisulfite conversion rate was on average 99% for all sample groups and consistent between the sample groups (S2 File). Principal component analysis (Fig 2A) and pairwise distance clustering of the samples (dendrogram/heatmap) (Fig 2B) revealed that the overall DNA methylation pattern distinguishes samples by generation and to a lesser extent by dietary group. It must be noted that livers were sampled at a different age of the zebrafish in F_0_ (98 DPF) and F_1_ (140–142 DPF). The F_0_ generation was clearly separated between the dietary groups although there were only three samples left in the control group as the other three samples did not meet DNA quality cut-offs for sequencing. The PCA plot shows a higher degree of overlap for the F_1_ generation compared to the F_0_ generation.

Among all samples, the number of common methylated loci was less for F_0_ than for F_1_ livers (Table 1). After filtering by a minimum read depth per locus of 10, and combining samples based on methylated loci common to all samples within a treatment comparison group (e.g. F_1_ high ARA vs F_1_ control), the remaining methylated loci were analysed for differential methylation between the dietary groups. We found that the number of DML between high ARA and control group was marginally higher in F_0_ livers (2338) than in F_1_ livers (2142). In addition, the number of hypermethylated (1091) compared to hypomethylated (1051) loci was almost identical in F_1_ livers, whereas F_0_ livers showed greater hypermethylation (1411) than hypomethylation (927). We found no difference between high ARA and control livers when comparing total CpG methylation rates, both groups showed on average 85% methylation (S2 File).

**Table 1:** Total methylated loci before and after filtering, number of differentially methylated loci (DML) and hyper- and hypomethylated loci in F_0_ and F_1_ livers following a high ARA diet in F_0_.

	Total methylated loci	Total methylated loci after filtering ^1^	DML ^2^	Hyper-methylated loci	Hypo-methylated loci
F_0_	1 323 478	491 007	2 338	1 411	927
F_1_	1 584 128	790 735	2 142	1 091	1 051

^1^ Minimum read coverage ≥10 reads.

^2^ Methylation difference ≥25% (q-value ≤0.01) of high ARA compared to control group.

#### Differential methylation across genomic regions

**Fig 3:**
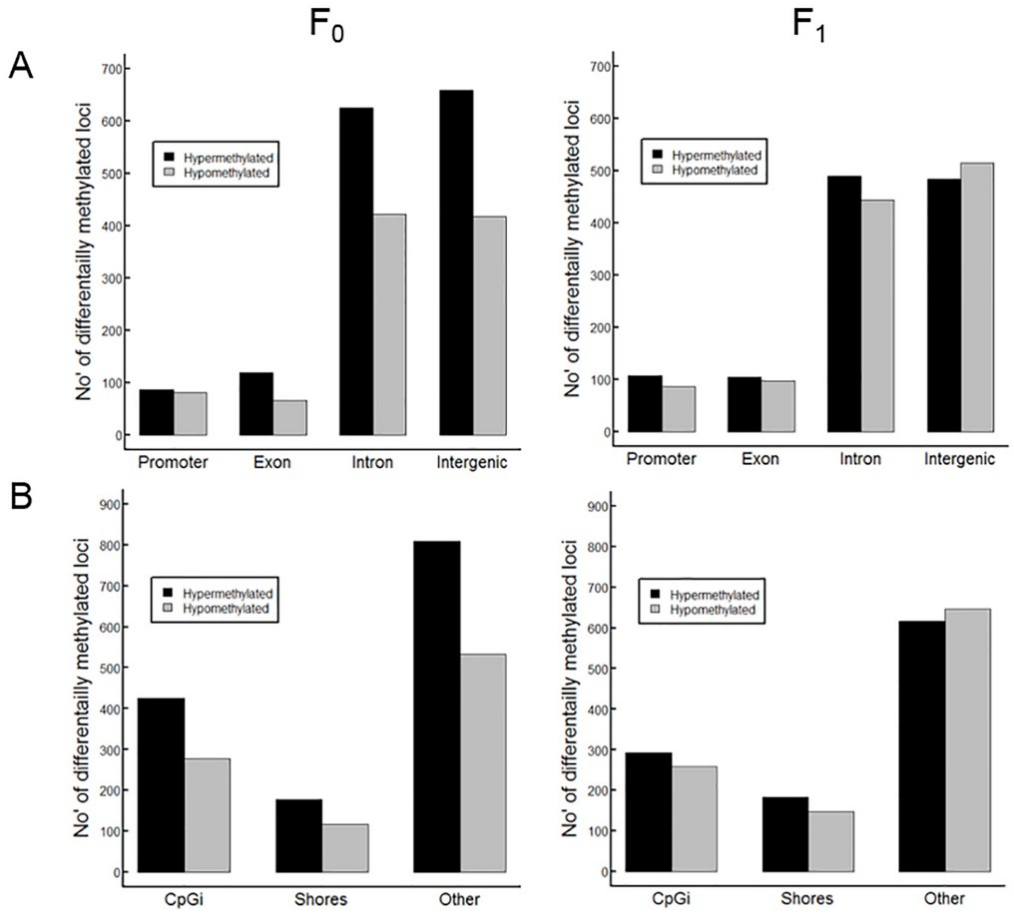
Differentially methylated loci across genomic regions in F_0_ and F_1_ livers. Differential methylation here shown as hyper- and hypomethylated loci across promoters, exons, introns and intergenic regions (A), CpG islands (CpGi) and shores (B).

The distribution of DML was investigated in promoters, exons, introns, and intergenic regions (Fig 3A) to verify if the DML were randomly distributed or specifically enriched in specific locations of the genome. The results show that the overall DML distribution across genomic regions was similar between F_0_ and F_1_ livers, with fewer DML in promoters and exons compared to DML in introns and intergenic regions. However, the increased number of hypermethylation within the F_0_ group is predominantly in the introns (625) and especially in the intergenic regions (658) compared to F_1_ (introns: 490, intergenic: 483). In addition, exons of F_0_ have proportionally more hypermethylation (119) than hypomethylation (65) compared to F_1_ (hypermethylation: 104, hypomethylation: 97).

Searching for DML in CpG islands and CpG shores, we noticed increased differential methylation in CpG islands (F_0_: 702, F_1_: 551) than in the CpG shores (F_0_: 294, F_1_: 328) for both generations (Fig 3B). Again, we found that the F_0_ generation had more hypermethylation especially in the CpG islands (425) than F_1_ (292). However, the majority of DML fell outside the CpG island and CpG shore annotation categories, into other genomic regions (F_0_: 1342, F_1_: 1263), where again F_0_ high ARA livers show noticeably more hypermethylation (809) and less hypomethylation (533) than F_1_ (617/646). In terms of enrichment and depletion of DML within genomic regions, for the F_0_ cohort only CpG islands were significantly depleted with no significant difference found within CpGi shores, exons, introns and promoters. For the F_1_ cohort, all genomic regions were significantly enriched or depleted for DML, with CpG islands and introns depleted and CpGi shores, exons and promoters enriched for DML (S3 File).

#### Functional annotation of DML

We associated DML within promoters and gene bodies with specific biological pathways (S5 File). No KEGG pathways and only one GO term (proteinaceous extracellular matrix) was significantly enriched (q-value cut-off <0.05) in F_0_, whereas in F_1_ no significantly enriched terms were identified.

Among the most differentially methylated loci between the dietary groups in F_1_ livers, *crebbpb* was dominating with three hyper- and one hypomethylated DML (S4 File). *crebbpb* codes for the nuclear coactivator cAMP-response element-binding protein (CREB) binding protein that plays a key role in various signaling pathways through interacting with numerous transcription factors.

#### Common F_0_ and F_1_ DML

DML from high ARA vs. control group analysis in both generations were merged to find common loci between the F_0_ and F_1_ methylation differences (S4 File). In total, 190 DML assigned to promoter, gene body and intergenic regions were common between F_0_ and F_1_ DML. Among them, 5 DML exclusively assigned to promoters were at identical sites in F_0_ and F_1_ following both same (*cryabb* and *nup160*) and opposite methylation patterns (*rsf1b*.*1*, *si*:*dkey−4c2*.*11* and *zbtb24*) between the generations. For *cryabb*, *nup160* and *zbtb24* that have one assigned DML in F_0_ livers, at least two or more DML have been associated to same gene showing same methylation patterns in the F_1_ livers (S4 File). Among the genes, *cryabb* (crystallin alpha B) assigned to four F_1_ DML codes for a small heat shock protein that is involved in the stabilization of stress-related cellular processes such as cell cycle, differentiation, apoptosis and redox homeostasis.

### Differential methylation and gene expression

#### Genes linked to differential methylation and differential expression

**Fig 4:**
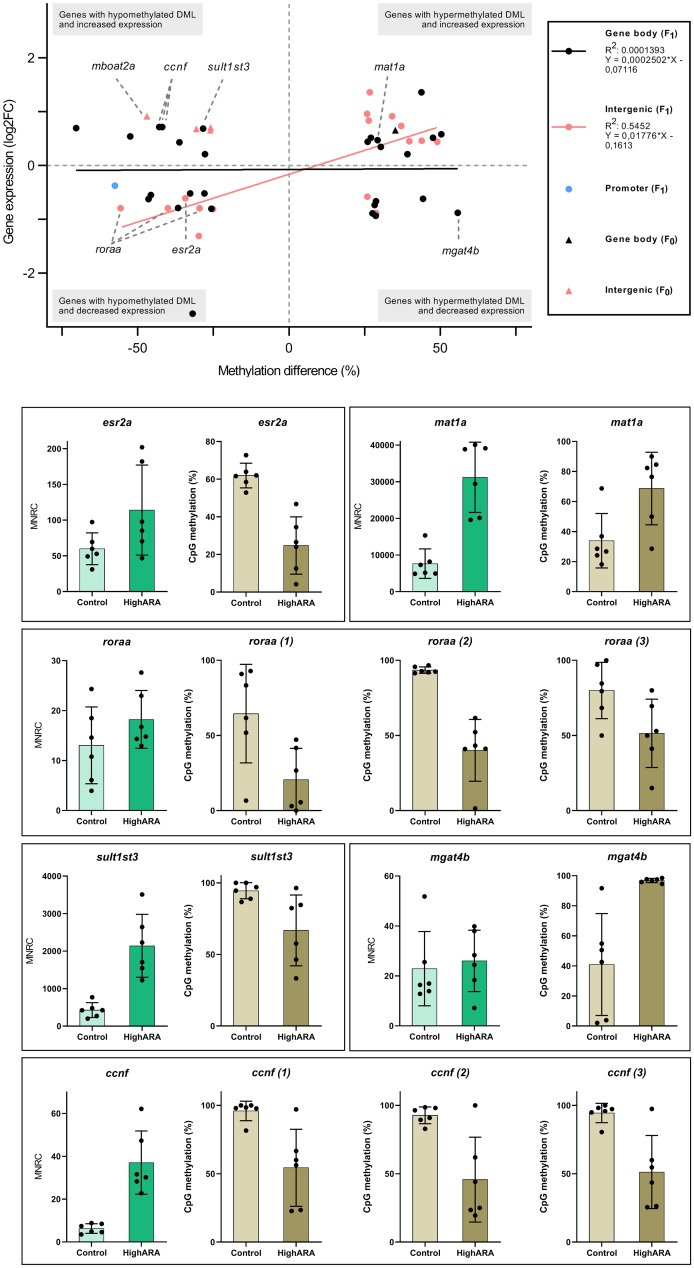
Genes associated with differential methylated loci (DML) and differential expression in F_0_ and F_1_ livers. **A:** Plot showing genomic locus-specific methylation differences and gene expression of concordant genes in F_0_ (5) and F_1_ (37) livers. Differentially expressed genes (adjusted p <0.05 for F_1_ and p <0.1 for F_0_) and DML (methylation difference +/- ≥25% and q-value ≤0.01) were obtained from F_0_ and F_1_ livers comparing high ARA and control group. Some of the genes in F_1_ were annotated to more than one DML, which led to 46 comparisons in total (S8 File). A best fit line with equation and goodness of fit (R^2^) quantified by linear regression (S8 File) is shown for F_1_ DML located in gene bodies and intergenic regions. **B:** Bar graphs showing individual read counts and individual CpG methylation levels of single genes tagged in Fig 4A for each replicate of the high ARA and control group. Gene expression is shown as mean normalized read counts (MNRC) in high ARA versus control livers. CpG methylation was calculated as the percentage of total methylated CpGs over the total number of CpGs assessed.

We searched for a connection between DML (S4 File) and DEG (S6 File) for F_0_ and F_1_ generation. We found 5 concordant genes between DML and DEG in F_0_ and 37 concordant genes in F_1_ (Fig 4A and Table 2). All overlapping genes for both generations are listed in S7 File.

**Table 2:** Genes linked to differentially methylated loci (DML) concordant with differentially expressed genes (DEG) in F_0_ and F_1_ livers due to inclusion of high ARA in the parental (F_0_) diet.

Generation	# Concordant genes to DML and DEG	Concordant gene symbols to DML and DEG
F_0_	5	*phldb2a*, *CABZ01052815*.*1*, *si*:*ch1073-189o9*.*1*, *mboat2a*, *fam20a*
F_1_	37	*magi1b*, *mgat4b*, *col14a1a*, *sult1st3*, *oxsr1b*, *phkg1a*, *slc4a4b*, *nek7*, *wdr62*, *abca12*, *crtc1b*, *si*:*ch211-194p6*.*12*, *si*:*dkey-10p5*.*10*, *gne*, *polr3h*, *ccnf*, *zgc*:*77086*, *sema3fb*, *park7*, *rpia*, *hykk*.*2*, *filip1b*, *roraa*, *si*:*dkey-248g21*.*1*, *esr2a*, *elac2*, *slc38a3b*, *add1*, *rps17*, *tomm70a*, *nrxn3a*, *cxxc5b*, *mat1a*, *lpar2a*, *prpf40a*, *CABZ01079024*.*1*, *slc26a2*

Plotting methylation differences against gene expression in F_1_, we found 29 overlaps (corresponding to 24 genes due to several DML annotated to the same gene) between DML assigned to a gene body and DEG showing no significant correlation between methylation (hyper/hypo) and gene expression (up/down). 16 overlaps (corresponding to 14 genes) involving F_1_ DML assigned to intergenic regions were significantly and positively correlated as shown in Fig 4A (p = 0.0011). Correlation and linear regression analysis is reported in S8 File. For single overlapping F_1_ genes flagged in Fig 4A, individual read counts (RNA-seq) and individual CpG methylation (RRBS) for each of the replicates in high ARA and control group are shown in Fig 4B. Excluding uncharacterized genes, DML associated with an F_1_ locus showing the greatest hyper- or hypomethylation were *mgat4b* (mannosyl (alpha-1,3-)-glycoprotein beta-1,4-N-acetylglucosaminyltransferase, isozyme B) and *roraa* (RAR-related orphan receptor A, paralog a), respectively.

Three hypomethylated loci in F_1_ were associated with the closest gene, *roraa*, which encodes a nuclear hormone receptor involved in lipid regulation. *roraa* was significantly downregulated in F_1_ high ARA livers. *esr2a* (estrogen receptor 2a), involved in retinoid signalling, was found as the closest gene to a hypomethylated locus in F_1_. *esr2a* showed significant downregulation in F_1_ high ARA livers. A hypomethylated locus in F_1_ overlapped with *sult1st3* (sulfotransferase family 1, cytosolic sulfotransferase 3), which functions in lipid homeostasis. *sult1st3* showed significant upregulation in F_1_ high ARA livers. One of the key genes in the methionine cycle, called *mat1a* (methionine adenosyltransferase I, alpha), was overlapping with a hypermethylated locus in both F_0_ (28% increased methylation) and F_1_ (29%). *mat1a* was significantly upregulated in F_1_ high ARA livers compared to the control, but not differentially expressed in F_0_. Three hypomethylated loci were overlapping with *ccnf* (cyclin F) that was upregulated in F_1_ livers.

One of the overlapping F_0_ genes was *mboat2a* (membrane bound O-acyltransferase domain containing 2a), which is involved in lysophospholipid metabolism. A hypomethylated locus was associated with *mboat2a* as the closest gene and *mboat2a* was upregulated in F_0_ livers.

#### Upstream regulators

Upstream regulators affecting downstream biological functions were predicted using IPA based on F_1_ DEG. Among the 399 predicted upstream regulators, 44 showed an overlap with the F_0_ and F_1_ list of DML linked to a gene annotation (S9 File). After filtering, the cannabinoid receptor 1 (CNR1) had the strongest positive z-score of the upstream regulators predicted by IPA (S9 File). *cnr1* was overlapping with a hypomethylated locus in F_1_. However, *cnr1* was not among the DEG as it was filtered out prior to differential expression calling due to low read count in the sequenced livers. The majority of the upstream regulators were transcriptional regulators (PPARGC1A, NCOA2) and nuclear receptors (PPARD, RORA, PPARA, ESR2, NR0B2) involved in the regulation of fatty acids, lipids, estrogen and energy metabolism.

## Discussion

In the present study, we have shown that feeding the parents a diet high in ARA, an n-6 PUFA, can alter the DNA methylation patterns in livers of both the parents and their adult progenies. This demonstrates that the epigenetic DNA methylation pattern is sensitive to dietary ARA levels over generations. We reported previously, that a diet high in ARA did not alter the growth of adult zebrafish in either parents or progeny [47, 48]. Earlier studies have shown that nutrition can influence DNA methylation, which affects phenotypes [5, 66, 67]. To our knowledge, this is the first study showing that changing the fatty acid profile in the parental diet can affect DNA methylation at locus-specific sites in zebrafish liver.

DNA methylation is the most studied epigenetic mechanism, which is known to regulate gene expression potential. When comparing the DNA methylation pattern and gene expression profiles in mature zebrafish livers in this study, it was hard to find a consistent pattern between direction of methylation and changes in gene expression. In general, we observed a higher degree of overlap between methylation and transcription profiles in the F_1_ generation than in the F_0_ probably due to a greater number of differentially expressed genes detected in F_1_. In response to the ARA fortified diet, we observed a larger distribution of DML in introns and intergenic regions than in exons and promoters for both generations. However, the latter was a significantly enriched region in F_1_ livers, meaning more DML detected than expected. A latest study [5] examined DNA methylation in livers of zebrafish fed a micronutrient-modified diet, also reported similar findings, with promoters enriched for DML. Methylation in promoters is widely believed to alter gene expression [68] though methylation outside promoters has also been shown to play a role in gene regulation [69]. Although not differentially expressed, *crebbpb* is one gene that was assigned to four among the most differentially methylated loci present in either an exon or an intron in the F_1_ livers. CREBBP is playing critical roles in embryonic development, growth control, and homeostasis by coupling chromatin remodeling to transcription factor recognition [70, 71]. A previous zebrafish study suggested that methylation associated with promoters was not the major determinant of transcription when comparing methylation and transcriptional data [72]. This is in line with our results, which showed relatively few promoter region associations between DML and DEG. Regardless, we reported interesting patterns among promoter DML that are identical between F_1_ and F_0_ DML (5 DML), where e.g. two identical DML show the same methylation pattern in both generations. Interestingly, those genes were assigned to one F_0_ DML and at least two or more F_1_ DML showing same methylation patterns. One of them we reported was *cryabb* that functions in skeletal muscle tissue development, repair and stabilization of stress-related cellular processes such as cell cycle, differentiation, apoptosis and redox homeostasis [73, 74]. Whether some of the reported methylation differences across different genomic regions are due to random chance and others due to the dietary treatment in this study, remains unanswered. Although the number of total DML were similar in both generations of the present study, a slightly higher number of hypermethylated DML were observed in the parental F_0_ compared to the F_1_. A general increase in genome methylation following ARA exposure has previously been shown in mammalian cells [45]. As noted by us and by others using rodents [75], DNA methylation patterns are sensitive to both parental (previous) and within generation environmental changes. This highlights nutrition as important modulator of DNA methylation pattern changes, as earlier implied for vitamins by others [76].

Several genes associated with F_0_ and F_1_ DML overlap between the generations, however, no significantly enriched pathways were observed for either F_0_ or F_1_ DML except from one GO term in the F_0_ generation. Despite few overlaps between DML and DEG in F_0_, we associated DML with the metabolic profiles reported previously [47]. The F_0_ metabolic profiles of juvenile zebrafish from the present study showed that high dietary ARA induced both inflammatory and anti-oxidative responses affecting lipids and amino acids [47]. Interestingly, *mboat2a* was one of the overlapping genes between DML and DEG in the F_0_ generation. This gene functions in lysophospholipid metabolism, which is consistent with the reported shift in lysophospholipid profiles for F_0_ fish fed high ARA levels [47]. However, surprisingly few concordant genes to F_0_ DML and DEG were detected. Many factors might have masked some differences in the F_0_ generation leading to few overlaps for instance general low differential gene expression, plasticity of DNA methylation [77, 78], age [79] and variation introduced by the cell type specific nature of DNA methylation [80].

An intriguing finding was the significant positive correlation between intergenic DML and DEG in offspring livers (and no correlation between gene body DML and DEG). Genes annotated to intergenic DML were based on the nearest gene TSS to the DML, which varied between a few thousand to hundreds of thousands of bases distant. Regulatory elements, such as promoters, enhancers, silencers, noncoding RNA, etc, can be many thousands of bases distant from the genes. They often have a considerable and complex combinatorial effect on gene expression and phenotype [81]. We need to stress that though we found a significant correlation between DML and DEG in intergenic regions, this is still based on a relatively small number of overlaps (n = 16) and we have not identified any causal links. That we found a positive correlation, i.e. hypermethylation correlating with upregulation (e.g. solute carriers 38a3b and 26a2) and hypomethylation correlating with downregulation (e.g. *roraa and esr2a*), was surprising, given that the focus of functional methylation has been primarily on promoter regions, where hypermethylation has been shown to functionally repress gene expression [82]. However, the functional associations between gene expression and methylation in different genomic regions (such as gene bodies) is still poorly understood [83, 84]. We have reported the neighboring genes and associated intergenic DML as a reference for further functional exploration. It is possible that future studies will identify functionally associated regulatory elements using the DEG associated DML we have reported here.

DNA methylation changes might control gene expression at early developmental stages and in specific tissues that can prime gene expression changes later in life controlling specific pathways [30, 85, 86]. In the present study, we found several genes with parental diet-associated methylation changes that also showed differential gene expression in the mature progeny livers. We found no significant pathway enrichment for F_1_ DML, but interestingly some of the F_1_ DML genes related to pathways previously reported to be affected by ARA at both a transcriptional and a metabolic level [47, 48]. Of the genes overlapping between DML and DEG in F_1_, some function in the methionine cycle (*mat1a*), lipid (*roraa*, *sult1st3*, *lpar2a*, *abca12*) and estrogen signalling (*esr2a*). In the present study, we looked for possible upstream regulators based on the F_1_ DEG and if some of them could explain the observed differences in both DNA methylation and gene expression. Several of the upstream regulators predicted by IPA were linked to DML, of which most were transcriptional regulators and nuclear receptors regulating fatty acid, lipid, estrogen and energy metabolism. Another gene, cannabinoid receptor 1 (CNR1), with an associated DML was suggested as the most activated upstream regulator in the progeny livers. Cannabinoid receptor 1 gets activated by endocannabinoids in liver, affecting *de novo* lipogenesis and fatty acid catabolism [87]. Remarkably, we observed increased endocannabinoid levels in F_0_ fish fed the high ARA diet [47], but no causal relationship has been found.

We showed that the overall changes in methylation were bigger in between generations and to a lesser extent between dietary groups. This can be attributed to natural occurring variation across generations representing a covariate and differences in the age of the samples analyzed for DNA methylation [79]. Based on the few associations we made between the methylation and transcription changes in grown offspring, it is also difficult to conclude on whether parental (F_0_) diet influenced the environment of the developing embryo while priming hepatic gene expression of progeny [18, 33, 88]. Two studies in rodents could not link parental diet-associated DNA methylation changes with gene expression in the progeny [44, 89]. This underlines the need for more detailed knowledge on the complex link between nutrients and epigenetic modifications, for example, in the germline and early embryo resulting in altered gene expression and metabolic phenotypes in grown offspring [88]. Nevertheless, we reported several DNA methylation differences present in the livers of grown offspring. Although we could not link overall methylation differences to DEG in the liver, it is conceivable that the reported methylation changes rather regulate gene expression during early development or in other tissues. This indicates that epigenetic mechanisms of gene regulation may act in a spatio-temporal matter, meaning tissue-specific and specific for developmental stages. More studies are needed to further illuminate and validate the mechanisms.

## Conclusions

This study has shown that parental dietary ARA influences DNA methylation in zebrafish liver. Hepatic methylation patterns across different genomic regions have been reported in two generations. We identified 2338 loci in the parental livers and 2142 loci in the livers of their progeny showing differential methylation between the dietary groups. We compared the DNA methylation changes in progeny livers to existing gene expression changes, but only based on few gene annotations. Although we reported several genes possibly regulated by diet-associated methylation changes, our results are limited to liver tissue, and causal or functional associations remain undiscovered. Thus, it is possible that the relation between DNA methylation and gene expression changes is stronger during early developmental stages or in tissues other than liver. The effects of nutritional induced DNA methylation changes at specific CpG loci in a transgenerational context and the extent of epigenetic gene regulation need to be verified by further studies.

## Supporting information

S1 File Ingredients, nutritional and selected fatty acid composition of control and high ARA diet. (PDF)

S2 File Global methylation means, bisulfite conversion and mapping rates from reduced representation bisulfite sequencing of F_0_ and F_1_ livers. (XLSX)

S3 File Log2 transformed enrichment ratios for differentially methylated (DM) loci within genomic regions (CpG islands, CpG island shores, exons, introns and promoters) in F_0_ (A) and F_1_ (B) zebrafish liver. (PDF)

S4 File F_0_ and F_1_ differentially methylated loci annotation (Ensembl) by genomic regions and identical differentially methylated loci between generations. (XLSX)

S5 File Functional annotation of F_0_ and F_1_ differentially methylated loci for KEGG pathways and GO terms. (ZIP)

S6 File Differentially expressed genes from RNA-sequencing of F_0_ and F_1_ livers comparing control and high ARA group (Ensembl). (XLSX)

S7 File Common gene annotations to differentially methylated loci (DML) and differentially expressed genes (DEG) in F_0_ and F_1_ zebrafish livers. (PDF)

S8 File Differential methylation and differential gene expression of overlapping genes from F_1_ and F_0_ generation comparing high ARA and control group. (XLSX)

S9 File Predicted upstream regulators based on differentially expressed genes in F_1_ generation using IPA. (XLSX)
