# Supplementary material for: Profiling DNA methylation patterns of zebrafish liver associated with parental high dietary arachidonic acid
Source: PLoS One. 2019 Aug 9;14(8):e0220934. doi: 10.1371/journal.pone.0220934 (PMC6688801; doi:10.1371/journal.pone.0220934)
Supplement: S1 File — (PDF) [file pone.0220934.s001.pdf]

S1 File. Ingredients, nutritional and selected fatty acid composition of control and high ARA diet.
Ingredients (A), dietary fatty acid profiles (A) and nutritional composition (B) have been previously reported: **A**: Adam AC, Lie KK, Moren M, Skjaerven KH. *High dietary arachidonic acid levels induce changes in complex lipids and immune-related eicosanoids and increase levels of oxidised metabolites in zebrafish (Danio rerio).* Br J Nutr. 2017 May 09:1-11. **B:** Adam AC, Skjaerven KH, Whatmore P, Moren M, Lie KK. *Parental high dietary arachidonic acid levels modulated the hepatic transcriptome of adult zebrafish (Danio rerio) progeny*. PLoS ONE. 2018; 13(8):e0201278.

	Control diet	High ARA diet
**Ingredients (g/kg DM)**		
Protein blend *	767.9	767.9
Agar †	1.0	1.0
Fish oil ‡	8.0	8.0
Rape seed oil §	48.0	20.0
Flax seed oil §	20.0	4.0
Cargill´s ARA-rich oil ‖	4.0	48.0
Dextrin †	46.17	46.17
Cellulose ¶	19.3	19.3
Lecithin **	20.0	20.0
Mineral mix ††	50.0	50.0
Vitamin mix ‡‡	10.0	10.0
Methionine §§	2.5	2.5
Cyanocobalamin (1%) ‖‖	0.99998	0.99998
Folic acid (97%) ‖‖	0.0111	0.0111
Pyridoxine hydrochloride ‖‖	0.0199	0.0199
Astaxanthin ¶¶	0.3	0.3
Sucrose †	1.0	1.0
Tocopherol mix ***	0.75	0.75
**Nutritional composition**		
Lipid (g/100g ww)	12.2	12.9
Protein (g/100g ww)	50	50
Ash (g/100g ww)	6.8	7.0
Energy (J/g ww)	21100	21400
**Selected fatty acids (mg fatty acid/g diet)**		
18 : 1n-9 oleic acid	43.32	27.24
18 : 2n-6 linoleate (LA)	31.27	26.80
18 : 3n-3 α-linolenate (ALA)	17.50	6.43
18 : 4n-3 stearidonate	0.25	0.25
20 : 3n-6 dihomo-linolenate	0.17	1.79
20 : 4n-6 arachidonate (ARA)	1.87	20.66
20 : 5n-3 eicosapentaenoate (EPA)	1.26	1.3
22 : 4n-6 adrenate	0.05	0.14
22 : 5n-6 docosapentaenoate (n-6 DPA)	0.05	0.04
22 : 5n-3 docosapentaenoate (n-3 DPA)	0.15	0.15
22 : 6n-3 docosahexaenoate (DHA)	1.42	1.37
Sum unidentified	1.27	1.55
Sum identified	121.00	118.00
Sum saturated	17.40	25.20
Sum monounsaturated	49.30	31.90
Sum polyunsaturated	54.60	60.50
Sum EPA + DHA	2.68	2.67
Sum n-3 PUFA	20.90	9.75
Sum n-6 PUFA	33.60	50.70
(n-3) / (n-6)	0.6	0.2

Data are expressed as mean of two technical replicates. ARA, arachidonic acid.
* BioMar AS products: fish meal, 5 %; krill meal, 1 %; soya protein concentrate, 6.2 %; maize, 5 %; wheat, 7.5 %; wheat gluten, 13 %; pea protein, 49.8 %; field peas, 12.5 %.
† Dissolved in 200 ml heated Milli-Q water, Sigma Aldrich Norway AS.
‡ Cod liver oil; Møllers, Axellus AS.
§ Rømer Produkt.
‖ Donated by Cargill (40 % ARA, Alking Bioengineering).
¶ Sigma Aldrich.
** Alfa Aesar.
†† Merck; ingredients (g/kg of diet): $CaHPO_4$ x $2H_2O$, 30; $CoCl_2$ x $6H_2O$, 0.007; $CuSO_4$ x $5H_2O$, 0.02; K_2SO_4, 15; KI, 0.05; $MgSO_4$ $7H_2O$, 5; $MnSO_4$ x H_2O, 0.05; NaCl, 2.873; Se-yeast, 0.2; $ZnSO_4$ x $7H_2O$, 0.5; $FeSO_4$ x $7H_2O$, 0.6.
‡‡ Obtained from Vilomix Norway AS, Norway; without cyanocobalamin, folic acid and pyridoxine hydrochloride (vitamin B_6) because of the trial set up with two directions (mg/kg of diet): vitamin A, 20; vitamin D, 4; vitamin E (50 %, acetate), 200; vitamin K (50 %), 10; vitamin C (35 %, phosphate), 350; choline, 1000; ascorbic acid, 1000; thiamine hydrochloride, 15; riboflavin (80 %), 19; nicotinamide, 200; inositol, 400; calcium pantothenate, 60; biotin (2 %), 50; filler (protein blend), 6672.
§§ Sigma-Aldrich.
‖‖ Normin AS.
¶¶ Dissolved in the agar solution; provided as a gift from G.O. Johnsen AS.
*** Provided as a gift from BASF.
