# Supplementary material for: Profiling DNA methylation patterns of zebrafish liver associated with parental high dietary arachidonic acid
Source: PLoS One. 2019 Aug 9;14(8):e0220934. doi: 10.1371/journal.pone.0220934 (PMC6688801; doi:10.1371/journal.pone.0220934)

S3 File. Log2 transformed enrichment ratios for differentially methylated (DM) loci within genomic regions (CpG islands, CpG island shores, exons, introns and promoters) in F_0 (A) and F_1 (B) zebrafish liver. Significantly enriched (i.e. positive y-axis values) or depleted regions (negative y-axis values) are indicated with an asterisk (**A**: CpGi p=8.87e-23; **B**: CpGi p=1.35e-34, CpGi shores p=0.0012, exon p=0.0024, intron p=0.0034, promoter p=2.35e-07). Significance ($p<0.05$) was determined using a hypergeometric test. Enrichment indicates that the ratio of DM loci to methylated loci within the genomic region is greater than the ratio of DM loci to methylated loci within the entire genome. Numbers within bars are total number of DM loci per region.

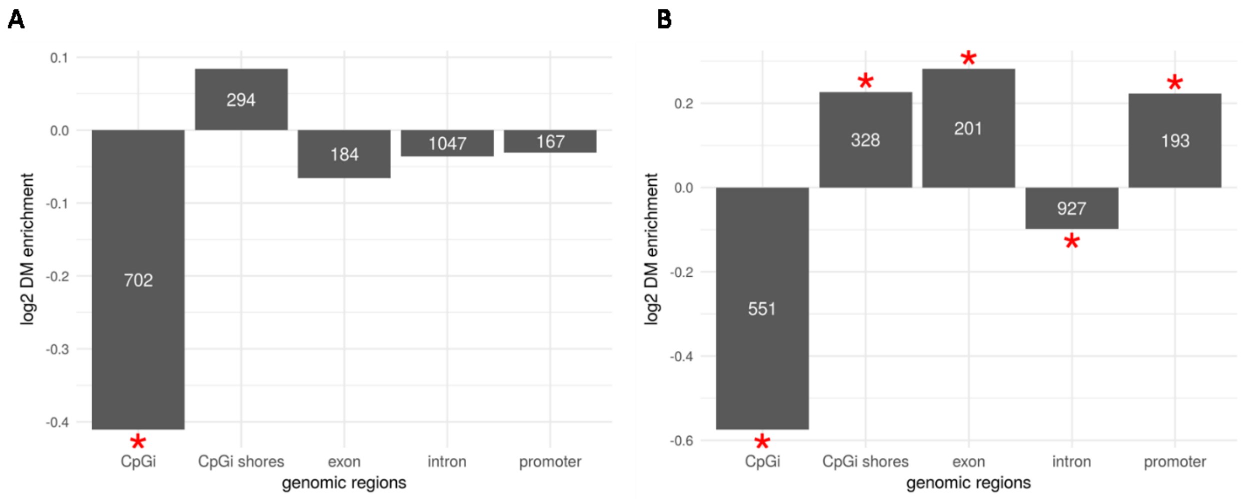

Supplement: S3 File — (PDF) [file pone.0220934.s003.pdf]
