# Supplementary material for: Profiling DNA methylation patterns of zebrafish liver associated with parental high dietary arachidonic acid
Source: PLoS One. 2019 Aug 9;14(8):e0220934. doi: 10.1371/journal.pone.0220934 (PMC6688801; doi:10.1371/journal.pone.0220934)
Supplement: S7 File — (PDF) [file pone.0220934.s007.pdf]

S7 File. Common gene annotations to differentially methylated loci (DML) and differentially expressed genes (DEG) in F_0 and F_1 zebrafish livers. Venn diagram underlies gene annotations to F_0 and F_1 DML with a methylation difference ≥25% (q-value ≤0.01) between high ARA and control group. DEG obtained from F_0 (adjusted p <0.1 cut-off) and F_1 livers (adjusted p <0.05 cut-off) comparing high ARA and control group using Venny 2.1 (Oliveros JC. Venny. An interactive tool for comparing lists with Venn's diagrams. 2007-2015; Available from: http://bioinfogp.cnb.csic.es/tools/venny/index.html.)

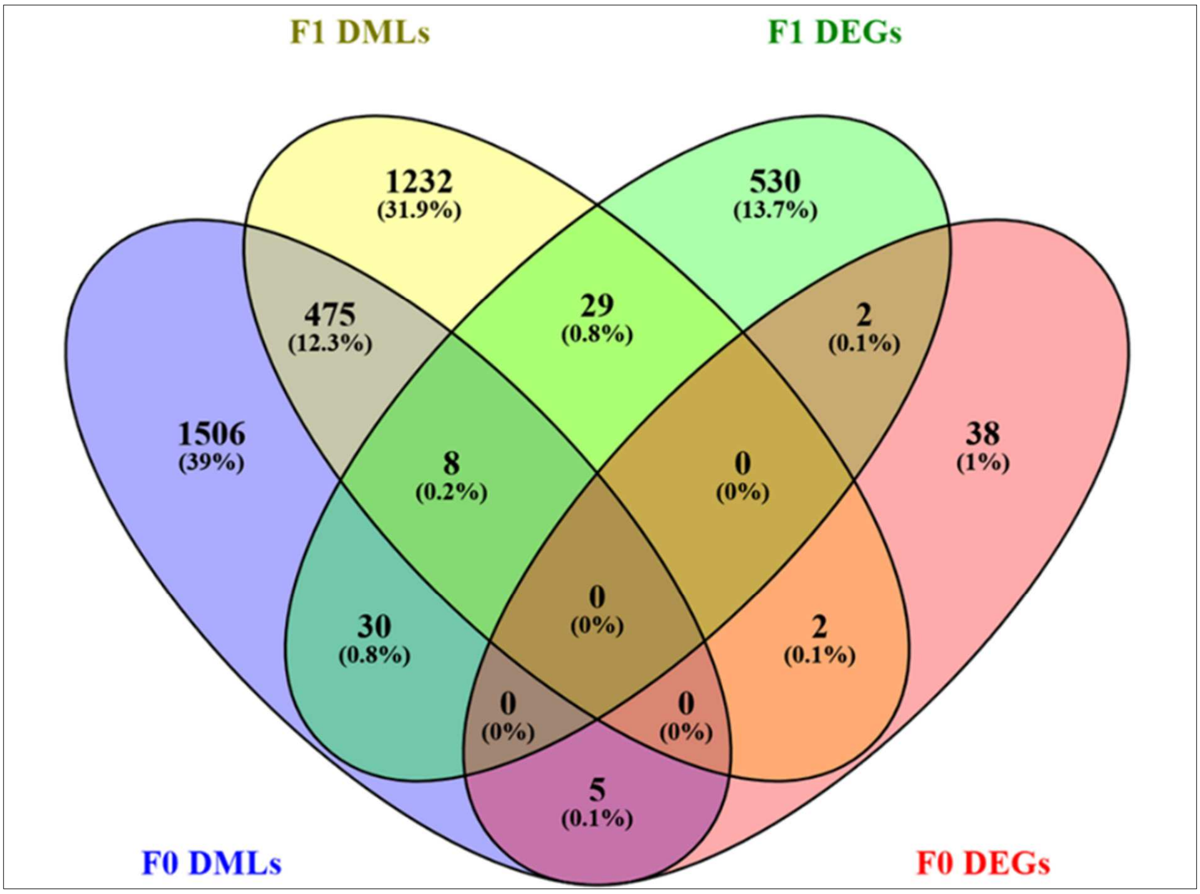

Data sets	Overlapping annotated genes	Gene symbol
F_0 DML and F_0 DEG	5 (0.1%)	phldb2a, CABZ01052815.1, si:ch1073-189o9.1, mboat2a, fam20a
F_1 DML and F_1 DEG	37 (1%)	magi1b, mgat4b, col14a1a, sult1st3, oxsr1b, phkg1a, slc4a4b, nek7, wdr62, abca12, crtc1b, si:ch211-194p6.12, si:dkey-10p5.10, gne, polr3h, ccnf, zgc:77086, sema3fb, park7, rpia, hykk.2, filip1b, roraa, si:dkey-248g21.1, esr2a, elac2, slc38a3b,

		add1, rps17, tomm70a, nrxn3a, cxxc5b, mat1a, lpar2a, prpf40a, CABZ01079024.1, slc26a2
F_0 DML and F_1 DML and F_1 DEG	8 (0.2%)	tomm70a, nrxn3a, cxxc5b, mat1a, lpar2a, prpf40a, CABZ01079024.1, slc26a2
F_0 DEG and F_1 DEG	2 (0.1%)	sec31a, glud1a
F_0 DML and F_1 DML	483 (12.5%)	tm9sf4, cd276, asic4b, grna, cyp4t8, ddx3b, mpped2a, lrp5, plxna3, cdh23, camta1b, pcdh15b, spred2b, kmo, f9a, hnrnpa1a, TENM2 (1 of many), col2a1b, marveld1, psmb3, rbfox1, cdkl5, eif4e2rs1, npas1, wnt10a, ncam2, zgc:158689, ncoa2, ntm, psmd3, dgkh, col12a1b, park2, plecb, ptk2ba, lpp, cacna2d4b, opn7a, pcbp4, MARK4 (1 of many), cpne8, nrp1b, si:ch211-106h11.3, CABZ01052588.1, adamtsl3, camkva, sult2st3, msra, nup188, tns3.2, usp36, pax2b, ipo7, gpd1c, plxnb2b, il1rapl2, igsf8, TRAPPC8, traf4b, trim35-9, pmepa1, dlg4b, loxl3b, cemip, neurod6a, fstl5, cadm4, spred1, tgfb1a, CABZ01066035.1, FRMD4A, kcnh5a, ms4a17a.7, KLHL29 (1 of many), xkrx, tp63, csmd1a, gdf11, ptprga, OTUD7A, si:dkey-14d8.20, apex1, b3galnt2, pcxb, gli3, fgfrl1b, plpp1a, EML5, got1l1, pparab, nucb1, capn8, npas4a, gfra4a, si:dkey-33c12.3, si:dkey-253d23.5, hck, trim46b, mcoln1b, soat2, sdr42e2, ca16b, sh3pxd2aa, cluhb, si:ch211-233a24.2, zeb2a, fgf12b, dhx37, dgkg, abcb7, p2ry10, fam46d, CABZ01081780.1, hecw2b, FO704712.1, mgat4a, si:dkey-32n7.7, kcne4, fam19a5a, adgrl2a, ghrh, zbbx, glis3, zgc:154058, crygm2d8, nbeaa, creb5b, furinb, cntnap5a, itpr1b, dzip1l, gas7a, rmdn1, KCNK12, lonrf1, neo1b, smad4a, arhgap32a, slc4a11, bmpr1bb, arl15a, znf1140, frem2a, fam198a, ano8b, znrf2a, si:ch211-236p22.1, NAV1 (1 of many), tex2, cspg4, hmox2b, tns1a, tusc3, lrfn5b, stard13a, PRRC2B, cep112, kif13ba, rapgef4, zgc:153681, plekhg6, nrg2b, atxn1l, crygm2d1, CABZ01072827.1, doc2b, mast1b, il17rel, si:ch73-367f21.5, fbln7, coro7, znf1042, si:ch1073-174d20.1, onecut2, crygm2d20, mvb12ba, mapkap1, ppp3r1a, iqsec3b, BX005477.1, si:dkey-19a16.1, BX957308.1, si:ch211-154e10.1, BX255915.2, CDPF1, FP102167.1, cul4a, si:ch211-227m13.1, si:dkey-4c2.11, CT573383.1, BX323807.2, si:dkey-234i14.6, lrp1aa, CR376776.1, BX890602.2, st8sia1, meis2a, pcdh15a, BX640576.1, FQ976914.1, si:zfos-2326c3.2, zmiz2, rptor, si:ch73-315f9.2, cyp2k6, ppp2r2bb, rnf213a, si:ch73-309g22.1, cnn3a, akap8l, mon2, si:dkey-199m13.7, PRKCA (1 of many), iqsec3a, LRRC52 (1 of many), arhgap11a, thumpd1, pik3r6a, znf1174, BX004768.1, ccdc40, slc8a2b, KYAT3 (1 of many), esrrb, ago4, FQ790208.2, slc18a3b, CABZ01058650.1, pdlim5a, col5a3b, gpr19, si:ch211-165e15.1, dusp16, btr30, GTPBP2 (1 of many), itih3b, neo1a, card14, usp43a, gramd2a, bmpr1ba, crebbpb, CLASP1 (1 of many),

	si:ch211-134a4.1, nlgn3a, psd3l, KCNT1 (1 of many), tenm4, erc2, nfkb1, adgrg4a, zgc:171727, znf511, papss1, rsf1b.1, zbtb24, nup160, pimr181, slc16a5b, CU210919.2, cryabb, pdcd4b, nox1, 5S_rRNA, AL845312.1, bet1, si:ch211-212o1.2, pde4a, calcoco1b, zgc:110204, spon1a, gria1a, slc35b2, mycn, kcnk5a, lbx2, pfkpa, psmc1a, CAPN2 (1 of many), zgc:92242, TPO, creld2, ndufs4, TMEM114, aldh1a3, st3gal1l2, igfbp5a, ldlrap1b, im:7160594, katnbl1, brd1b, rcvrna, opn3, pcp4a, yars2, col4a1, itih1, ints8, dhx15, CABZ01040556.1, slc25a38a, cplx1, CYTIP, cadm2b, psd2, SLC9A1, ppargc1a, ube2d4, GRB14, CABZ01041962.1, VSIR, IKZF2, tmem222a, zgc:174719, pex5la, ncoa6, ids, slc29a3, si:dkey-78p8.1, cblc, zgc:194281, C9orf172 (1 of many), rubcn, gnpat, FAM20C (1 of many), bcl2l11, nanos1, U4, dre-mir-218a-1, CABZ01032476.2, SNORA23, BX539325.2, cyth1a, etv5a, fam124b, egr3, ddhd1a, BX470083.4, BX537109.1, rpl34, CU571257.1, si:ch211-166i24.1, CABZ01046949.1, atp5g3a, BX248118.1, si:ch73-334d15.1, shox2, zdhhc5a, slc35f3b, nvl, wdr11, dia1b, rad9b, CR391921.1, gsc, si:ch73-379f5.5, si:ch211-93g21.1, si:dkeyp-113d7.10, CT027825.1, BX255915.1, fbxo15, BX908388.1, si:ch211-149a19.3, txndc5, psmd5, CT573344.2, BX005153.1, ms4a17a.5, dlgap4a, them4, hbegfa, pou3f3b, slc16a1a, tfap2c, CU695141.1, kynu, gdf3, tnfaip2a, BX322618.1, CR376838.1, CU570682.1, ppp6r3, dchs1a, gsg1l2a, BX005121.1, CR388029.1, CT737162.2, AL929435.1, CR790366.2, CR847830.3, si:ch211-149e23.4, si:dkey-90a13.10, BX248113.1, GSE1, BX901883.1, BX663503.2, CU929046.1, BX511132.1, BX537110.1, PDZD4 (1 of many), or130-1, FO818714.1, BX530032.1, nipbla, AL953858.1, sncb, si:ch211-51f19.1, echdc3, foxi1, CABZ01044277.1, stard13b, rps6kal, CABZ01078350.1, CR855379.1, lsm14ab, CABZ01039820.1, CU695223.1, CABZ01067761.1, BX324119.1, zdhhc18b, lmx1ba, FP085414.1, axin2, mascRNA-menRNA, sept9b, U1, CR388184.2, CU856516.1, pimr35, tmem129, si:dkey-191g9.7, socs3a, pmp22a, lmbrd1, TM4SF19, si:ch211-283l16.1, CABZ01065131.1, BX950873.1, rims2a, si:ch211-207l14.1, si:dkey-279j5.1, CABZ01032362.1, dnajb1a, fcer1gl, CR847998.1, lnpa, ap1ar, prpsap1, BX323453.1, abhd18, srfb, CDH22, PKN2 (1 of many), CR854963.1, samd1a, si:ch211-161h7.4, CABZ01112168.1, si:dkeyp-11e3.1, CT737196.1, vegfaa, ITIH4 (1 of many), CT033790.1, CABZ01111454.1, TMEM229A, rfx3, ddx52, col4a3bpb, pcmtd1, itk, pcdh10b, AL929185.1, BX247870.2, si:ch211-117a13.2, zgc:163040, BX005073.3, tomm70a, nrxn3a, cxxc5b, mat1a, lpar2a, prpf40a, CABZ01079024.1, slc26a2

The comparison underlies DML annotations from F_0 and F_1 livers with a methylation difference ≥25% (q-value ≤0.01) between high ARA and control group from differential methylation analysis of RRBS data, and DEG from F_0 (adjusted p <0.1 cut-off) and F_1 livers (adjusted p <0.05 cut-off) from gene expression analysis of RNA-sequencing data.
